# Temporal and Spatial Focusing in SPAD-Based Solid-State Pulsed Time-of-Flight Laser Range Imaging

**DOI:** 10.3390/s20215973

**Published:** 2020-10-22

**Authors:** Juha Kostamovaara, Sahba S. Jahromi, Pekka Keränen

**Affiliations:** Circuits and Systems Research Group, ITEE Faculty, University of Oulu, 90570 Oulu, Finland; sahba.jahromi@oulu.fi (S.S.J.); pekka.keranen@oulu.fi (P.K.)

**Keywords:** 3-D range imaging, pulsed time-of-flight, single photon detection

## Abstract

The relation between signal and background noise strengths in single-photon avalanche diode (SPAD)-based pulsed time-of-flight 3-D range imaging is analyzed on the assumption that the SPAD detector is operating in the single photon detection mode. Several practical measurement cases using a 256-pixel solid-state pulsed time-of-flight (TOF) line profiler are presented and analyzed in the light of the resulting analysis. It is shown that in this case it is advantageous to concentrate the available optical average power in short, intensive pulses and to focus the optical energy in spatial terms. In 3-D range imaging, this could be achieved by using block-based illumination instead of the regularly used flood illumination. One modification of this approach could be a source that would illuminate the system FOV only in narrow laser stripes. It is shown that a 256-pixel SPAD-based pulsed TOF line profiler following these design principles can achieve a measurement range of 5–10 m to non-cooperative targets at a rate of ~10 lines/s under bright sunlight conditions using an average optical power of only 260 µW.

## 1. Introduction

3-D range imagers have traditionally been used in applications such as mapping, surveying, civil engineering, inspection and quality control [1,2,3], but there has been a growing interest recently in using 3-D range imaging techniques in a wider field of applications. The development of an autonomous, driverless car obviously calls for high-speed environment-sensing techniques [4], and other potential applications are robotics, security, small vehicle guidance, e.g., Unmanned Aerial Vehicles (UAVs) and virtual reality/augmented reality (VR/AR). Uses have also been found for 3-D range imaging in consumer electronics (games), man-machine interfaces (e.g., gesture control) and machine control [5,6,7].

The current mainstream technology in 3-D imagers is scanning LIDAR based on a spinning polygon mirror or a rotating measurement frame that can measure distances simultaneously in as many as 64 planes with high measurement speed and precision [8]. This kind of scanning LIDAR can achieve high performance but at a relatively high price, partly due to the device’s complicated construction. On the other hand, many of the new applications would favor a 3-D range imager with solid-state realization, i.e., without any mechanically moving parts, since it is this which would pave the way for reduced costs and miniaturization [9,10,11,12,13,14,15,16,17,18,19,20,21,22,23].

One quite successful approach to solid-state 3-D range imaging is based on phase comparison techniques, in which the transmitter sends a continuous wave (CW) modulated laser beam and the per-pixel distances to the target are deduced from the phase of the received signal with a CMOS active pixel sensor (APS) [19,20,21,22,23]. This technology gives a high image pixel resolution (x-y), but unfortunately, the measurement range (z) is currently limited to only a few meters. Moreover, even in that case the transmitter requires a relatively high average optical power in the W range [21]. Thus, this concept seems not to be particularly well suited for mid- or long-range applications, which would obviously require even more optical power.

Another popular approach is to use single photon detection-based (SPAD) receiver techniques in 3-D range imaging. The measurement method can be based on phase comparison or on the direct pulsed time-of-flight technique (pTOF) [9,10,11,12,13,14,15]. The SPAD detector is basically a p-n junction which is reverse-biased above its breakdown voltage and, due to its simplicity, can also be realized in standard CMOS in the form of dense 2-D arrays [24,25]. The junction breakdown induced by the absorbed photon results in a large Volt-level signal with sub-ns time precision and can be detected with standard digital circuitry. Thus, contrary to avalanche photo detector (APD) arrays working in linear detection mode, no sensitive (even to electronic crosstalk) and complicated analogue amplifiers are needed. In addition to the high sensitivity, these properties make the SPAD-based receiver approach a very interesting option for a 3-D range image receiver.

Pulsed TOF techniques, which are used widely in LIDARs, are known to achieve a long unambiguous measurement range with high precision, even in the single shot measurement mode. It is also known that shortening the length of the laser pulse improves not only the precision of the LIDAR but also its signal-to-noise ratio, assuming that the average transmitter power remains constant [26]. When using a SPAD as the detector, the dominant noise in many practical applications comes from random detections induced by the background radiation [27]. From this point of view, shortening the laser pulse would also intuitively make sense, since the ratio of valid signal detections to random background detections would then increase within the pulse envelope. Perhaps less obviously, it is also generally advantageous to focus the transmitter energy in space. This would mean that in solid-state line profiling (a particular case of range imaging), for example, it would be advantageous to minimize the thickness of the laser fan, i.e., to maximize the irradiance on the target surface. In addition, it is in some cases advantageous to use a sequential block-based illuminator in 3-D range imaging instead of a flood illuminator, as explained in in more detail below [28].

The goal of this paper is to present the key properties of SPAD receiver-based 2-D and 3-D range imaging techniques using the pulsed TOF approach. A simplified theory is given that allows one to calculate the signal-to-noise ratio of this kind of a measurement with varying system parameters and background illumination conditions. The theory is expressed in a form that clearly demonstrates the usefulness of focusing the pulse energy in time (short intensive pulses) and space (a narrow laser fan or illumination in blocks). Several practical measurement cases carried out with an 8 × 256 element SPAD-based pulsed time-of-flight line profiler in varying illumination conditions are shown and discussed with regard to the given analysis.

The paper is organized so that Section 2 presents a brief theoretical introduction to SPAD-based pulsed TOF while Section 3 focuses on practical examples of measurements realized with a solid-state line profiler developed following the principles proposed here. A discussion on the results and a summary of the work are given in Section 4.

## 2. The SPAD Receiver-Based Approach to Pulsed TOF Range Imaging

### 2.1. Design Considerations

**Figure 1:**
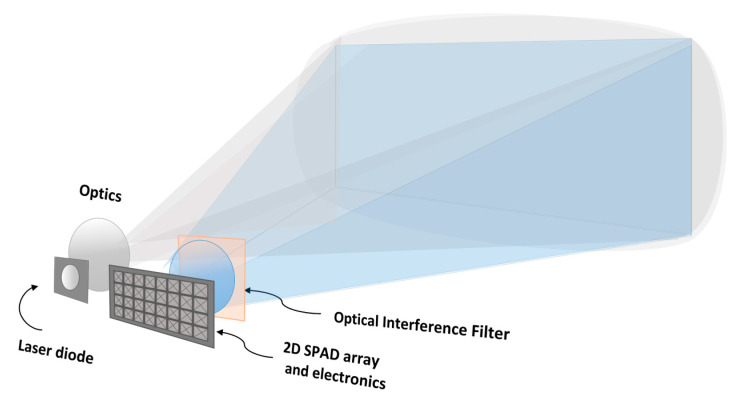
Basic functionalities of the SPAD receiver-based solid-state pulsed time-of-flight (TOF) 3-D range imager concept.

A block diagram of the basic functionalities of a pulsed TOF 3-D range imager utilizing SPAD receiver techniques is shown in Figure 1. The transmitter emits short, intensive laser pulses into the field-of-view (FOV) of the system. On the receiver side, a 2-D SPAD array with other relevant electronics (e.g., time-to-digital converters, TDCs) is located on the focal plane of the receiver optics. Thus, by measuring the transit times of the photons from the transmitter to the target surface and back to the receiver, a 3-D range image within the system FOV can be produced. In this 3-D image or point cloud, the x and y coordinates of a specific point on the target are generated by the position of the corresponding SPAD detector element and the z coordinate by the measured transit time. The arrangement in the example uses flood illumination (a flash imager), but, as already pointed out above, this is not necessarily the optimal arrangement and is used here for illustrative purposes only.

**Figure 2:**
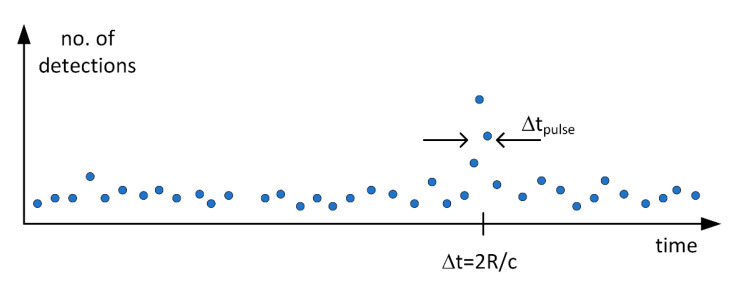
Illustrative result histogram for a single SPAD pixel yielded by a bunch of transmitted laser pulses. Δt_pulse_ is the width of the laser pulse (FWHM) and Δt the transit time of the pulse from the transmitter to the target and back to the receiver.

One specific feature of the above system is that in the case of a typical practical measurement, the probability of a single SPAD element detecting a signal photon at the limit of detection, i.e., at the maximum range, is << 1. This means that it is necessary to transmit a bunch of laser pulses in order to get a valid detection for each pixel (i.e., a high enough SNR). Thus, the measurement sequence of a 3-D image derived from a bunch individual laser pulses (e.g., 5000 pulses) is used to produce a histogram of single shot results for each pixel element (x,y), considering that there is mostly no detection for a single emitted pulse. The “noise” in the histogram will be produced predominantly by the random background hits and the “signal” by the signal hits at a time interval corresponding to the transit time of the photons to the target and back to the receiver, see Figure 2. The interesting question is obviously how the main histogram parameters, the strength of the signal and noise (and their filtered versions) are related to the relevant system parameters.

### 2.2. Signal Strength and Noise

Previous papers have discussed in detail the operation of a laser radar of this kind that uses photon counting statistics. The method can give accurate information on the detection and false alarm probabilities of the measurement setup [29,30,31]. We adopt here, however, a simplified approach that aims to give the key findings in form quite similar to that typically used when analyzing the operation of a pulsed TOF LIDAR working in the linear detection mode. In particular, we want to analyze the ratio between the signal and background radiation-induced noise strengths, SN_BG_R, which will give an indication of the performance of the measurement.

A very convenient parameter indicating the intensity of the background radiation is the mean time interval τ_BG_ between the detections introduced into a specific SPAD element. This can be calculated using Equation (1) and the relevant system parameters.

(1) $$\begin{array}{c} \tau_{\mathrm{BG}}=\frac{1}{\mathrm{PDP}}\left(\frac{E_{\mathrm{ph}}}{P_{B}}\right),\\ P_{B}\approx I_{S}\cdot A_{\mathrm{rec}}\cdot \rho \cdot {\left(\frac{{\mathrm{FOV}}_{\mathrm{SPAD}}\cdot \sqrt{\mathrm{FF}}}{2}\right)}^{2}\cdot BW_{\mathrm{opt}},{\mathrm{FOV}}_{\mathrm{SPAD}}=\frac{\Phi_{\mathrm{SPAD}}}{f_{\mathrm{REC}}} \end{array}$$

In Equation (1), P_B_ gives the total power of the background radiation seen by a single detector element in the SPAD array and τ_BG_ is the resulting mean time interval between the detections per pixel induced by the background radiation [27,32]. Here, I_S_ is the irradiance of background radiation on the target surface (i.e., caused by the Sun, ~700 mW/(m^2^ × nm) at ~810 nm), A_rec_ is the area of the receiver aperture, Φ_SPAD_ the diameter of the SPAD element, f_REC_ the effective focal length of the receiver optics, ρ the reflection coefficient of the Lambertian target, BW_opt_ the optical bandwidth of the receiver, E_ph_ the photon energy at the corresponding wavelength (2.5 × 10^−19^ J at ~810 nm), PDP the photon detection probability of a SPAD element (~4% at 810 nm in CMOS) and FF the fill factor of the SPAD detector element. FOV_SPAD_ is the linear field-of-view of the single SPAD element given in radians. The mean time interval induced by the background radiation may typically be from a few ns up to hundreds of nanoseconds depending on the system-level parameters and illumination conditions [27].

In addition to the background-induced noise, a SPAD also produces internally generated random dark counts. In a typical measurement scenario, i.e., outdoors or even indoors with lighting, this dark count rate (DCR) is nevertheless typically much lower than the background illumination-induced rate (BGR). More specifically, the DCR of a relatively small SPAD element (diameter <50 µm) realized in CMOS is typically less than 100 kHz, which corresponds to a mean time interval between counts of 10 µs, and has thus been neglected in the above analysis [25].

Assuming that the probability of a single SPAD element detecting a signal photon is << 1, SN_BG_R can now be given roughly as the ratio between the signal counts in any SPAD element of an array with x × y pixels and the square root of the number of random noise counts (the random component of N_BG_) observed during a time interval corresponding to the laser pulse width (the FWHM of the laser pulse is indicated by Δt_pulse_), see Equation (2). Thus, we have assumed that a simple running averaging filter matched to the laser pulse width is used here to filter the raw hit distribution. In Equation (2) this is indicated by the pulse width Δt_pulse_ that determines the time window during which the signal and noise counts are counted. For simplicity, we also assume that the transmitter power is evenly distributed across the illuminated area (or line). The above consideration of single photon detection regime holds for most practical cases when using laser diode as transmitters, at least at the limit of sensitivity, i.e., near the maximum range of the measurement system.

In Equation (2), M is the number of illumination blocks and thus M = 1 corresponds to the case of flood illumination. We will start the analysis from this assumption and later consider the case of block-based illumination. In that case, the available optical energy is directed to one of the M blocks (sub-FOVs) for a certain number of emitted laser pulses, and the system field-of-view (FOV) is then electrically sequentially scanned (e.g., in 16 blocks) to cover the whole system FOV. In Equation (2), f_LD_ is the pulsing frequency of the laser transmitter and f_frame_ is the desired 3-D range image frame rate. Thus, f_LD_/f_frame_ is the number of laser pulses emitted to achieve a valid image result, R is the distance to the target and Δt_pulse_/τ_BG_ is roughly the probability of detecting a noise count during a time interval corresponding to the laser pulse length.

(2) $${\mathrm{SN}}_{\mathrm{BG}}R\left(R\right)=\frac{N_{\mathrm{signal}}\left(R\right)}{\sqrt{N_{\mathrm{BG}}}}\approx \sqrt{e^{-\frac{2\times R}{\tau_{\mathrm{BG}}\times c_{\mathrm{light}}}}}\frac{\left(\frac{E_{\mathrm{opt}}\times \tau \times \rho \times A_{\mathrm{rec}}}{\pi \times R^{2}\times E_{\mathrm{ph}}}\times \frac{\mathrm{PDP}\times \mathrm{FF}}{\left(\frac{x\times y}{M}\right)}\times \frac{1}{M}\times \frac{f_{\mathrm{LD}}}{f_{\mathrm{frame}}}\right)}{\sqrt{\frac{1}{M}\times \frac{f_{\mathrm{LD}}}{f_{\mathrm{frame}}}\cdot \frac{{\Delta t}_{\mathrm{pulse}}}{\tau_{\mathrm{BG}}}}}\propto \frac{E_{\mathrm{opt}}\cdot \sqrt{M}}{\sqrt{{\Delta t}_{\mathrm{pulse}}}}$$

Thus, in essence, Equation (2) gives the ratio of the signal detections (nominator) and the square root of the total number of noise detections (e.g., detections from background and dark counts) within the pulse envelope, i.e., during Δt_pulse_ (denominator). It should be noted that the true signal-to-noise ratio of the measurement, SNR, differs from SN_BG_R, since the latter, as given in Equation (2), does not include the quantum noise of the signal itself. However, if the background-induced noise is dominant, as assumed above, SN_BG_R will give a rough approximation of the SNR as well at the limit of detection. In order to get the detection and false alarm probabilities the total SNR should be used, as discussed elsewhere [29,30,31]. The requirement for these depends on the specific application and is beyond the scope of this discussion. At a general level, an SNR of 5–10 is typically aimed at [33].

Some important conclusions can be reached based on Equations (1) and (2). It is clear that the most important single parameter affecting the system performance is the laser pulse energy. In addition, it is advantageous to concentrate the pulse energy in short pulses. In other words, the available average optical power, which may be limited for eye safety reasons or due to technical limitations (such as the laser driver capacity), for example, should be used in short, intensive pulses. This would improve the precision of the measurements, since the pulse envelope determines the uncertainty of the photon time position in the signal when in single photon detection mode. Moreover, as indicated in Equation (2), this would also improve the SN_BG_R (and SNR), since the number of noise counts at a given signal position is proportional to the laser pulse width (Δt_pulse_/τ_BG_ is reduced). Another point to be considered is that while increasing A_REC_ and PDP would increase the signal strength, this would also increase the amount of random shots induced by the background radiation (see Equation (1)), and thus their effect on the SN_BG_R will be proportional only to the SQRT of the increase.

As already pointed out above, M in Equation (2) is the number of illumination blocks, and M = 1 would thus hold for flood illumination. It is also possible, however, to use the average permitted illumination power in M blocks, i.e., to direct the available pulse energy to only one of the M blocks for a certain measurement period (even electrically, in a solid-state realization), i.e., for a certain number of emitted laser pulses. In this case, the FOV of the whole system should be electrically scanned in M separate steps. From the signal point-of-view, the total measurement time for a set number of required valid signal counts would be the same as in the case of flood illumination (assuming that the probability of a single SPAD element detecting a signal photon is << 1 even when block illumination is used). The number of laser pulses needed to get valid signal detections in a block is M times less due to the M times higher irradiance at the target (and thus M times higher detection probability), but this is compensated for by the need to scan over M blocks to obtain valid detections for the whole system FOV. On the other hand, the total number of noise detections in each of the SPAD histograms is now M times lower on average, which would improve the SN_BG_R in proportion to SQRT(M), albeit at the cost of higher transmitter complexity. This improved SN_BG_R can be used to speed up the measurement (by a factor of M), for example, or to increase the maximum measurement range (by a factor of M^1/4^). It is also important to note that from the average optical power point of view, the block illumination and flood illumination-based systems are equivalent. Thus, it is generally advantageous in SPAD-based pulsed TOF 3-D ranging to focus the pulse energy in terms of both time and space.

It is also important to note that the receiver electronics for a block-based illuminator can be simplified considerably, since the number of TDCs can be M times less than in the case of flood illumination. This advantage can be very important in practice, since the realization of a high performance TDC requires a considerable die area [28].

Another point to consider arises from the typical operation sequence of a SPAD detector. The breakdown induced by the detected photon needs to be quenched, either passively or actively, and this keeps the SPAD inoperative for a certain period after the breakdown. If maximum system efficiency is aimed at, the SPAD should be quenched actively, in which case the dead time will typically be some 10–20 ns [25]. If, however, a single SPAD element is allowed to detect only one photon during the operation cycle (i.e., the SPAD is activated only when a laser pulse is emitted), the probability of detecting a signal photon from the target distance is lowered by the fact that for some emitted laser pulses the detector may have been triggered by a random background illumination-induced photon before the signal photon had hit the detector. In other words, the signal photon can be detected only if no background photon has triggered the detector before the signal detection event. The probability of this occurring may be obtained from the Poisson distribution (e^−Δt/τBG^). Since this attenuation applies to both signal and background photons, the SN_BG_R is attenuated by the numerator square root exponential term given in Equation (2). One consequence of this is that the SN_BG_R is highly attenuated at longer measurement distances under high background illumination conditions (the blocking effect). In addition, the distribution of the number of measured background hits (as well as signal hits) decreases exponentially as a function of time and may need “gain compensation” in the histogram analysis [31,34]. This may also lead to a situation where an increase in the receiver efficiency (i.e., in A_rec_, PDP or FF) might actually reduce the SNR of the measurement, i.e., there is an optimum receiver aperture, for example, since any further increase in this would increase the attenuation factor more than the signal collection efficiency (due to a decrease in τ_BG_) [29,30,31].

## 3. Results of 2-D Line Profiling

### 3.1. System Description

**Figure 3:**
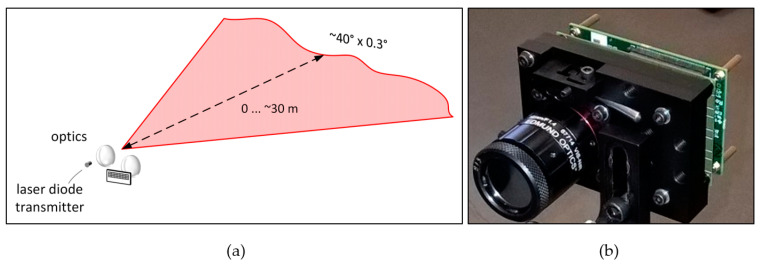
(**a**) Illumination principle and, (**b**) realization of the 2-D line profiler.

Motivated by the principles described above, a fully solid-state 2-D line profiler was developed, as shown in Figure 3 [31]. The transmitter is based on a custom-designed double heterostructure GaAs/AlGaAs quantum well (QW) laser diode working at ~810 nm and capable of producing laser pulses with an energy of ~2 nJ and width of 150–600 ps (FWHM) depending on the energy level [31]. The laser diode has a relatively large equivalent spot size (d_act_/Γ >> 1), which has been shown to enhance the gain switching effect, thus enabling high power sub-ns optical pulses without imposing strict requirements on the laser driver [35,36,37]. For ~2 nJ laser pulse energy, the drive current pulses have an amplitude of ~6 A and width of 1.5 ns, which can be produced relatively straight-forwardly with a FET driver [38,39]. The pulsing frequency of 130 kHz is limited by the receiver I/O. The cylindrical optics used to produce a horizontal laser fan with 37° × 0.3° opening angles are shown in Figure 3.

**Figure 4:**
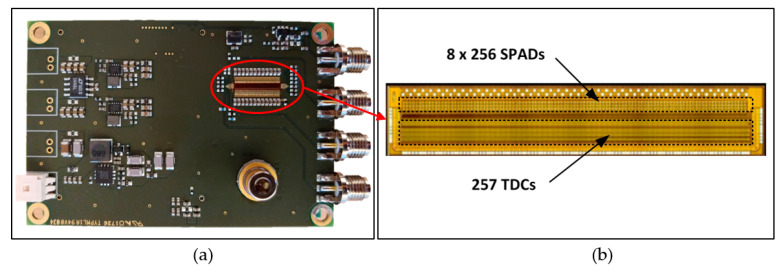
(**a**) Transmitter/receiver PC board and (**b**) the custom receiver CMOS IC, including 8 × 256 SPADs and 257 time-to-digital converter (TDC) circuits.

The heart of the system is a full-custom CMOS receiver IC, which includes on the same circuit die a SPAD array of 8 × 256 elements (unit element diameter ~40 µm) and 257 high-performance TDCs, Figure 4. The TDCs have a measurement range of ~100 ns and single shot precision of ~20 ps [40]. Eight parallel SPAD pixels share a single TDC element and any number of them can be connected to be active during the measurement. In addition, the SPAD elements can be set to be active with a delayed gate control (time gating) synchronized with the laser transmitter with a resolution of 20 ns. The diameter of the receiver optics is 11 mm and the focal length 16 mm. The width of the laser line on the receiver surface approximately matches that of the two SPAD detector lines. Note that this arrangement improves the SNR since the ratio of signal and noise detections per active pixel is maximized (as discussed above). Thus, during the measurement, all other SPAD lines are blocked out. On the other hand, the realization of the SPAD array with 8 lines of 256 elements simplifies the optomechanical adjustments. The optical bandwidth of the receiver is 20 nm. The line profiler achieves better than 10 mm single shot precision up to a range of 35 m with Lambertian targets given a measurement frame rate of 25 fr/s under low background illumination conditions. More details regarding the receiver circuit and system construction, and system-level test results, mostly in a laboratory environment, are presented in [31,40].

The line profiler includes an FPGA board (same size as in Figure 4), which transmits the measurement data (raw data histograms in all 256 SPAD/TDC channels) to a PC via a USB3 interface. The main functions of the PC software are to apply the gain compensation and filtering to the measurement histograms and to determine the target distances in all the channels for the result output (line profile) [31]. The key parameters of the developed line profiler are summarized in Table 1.

### 3.2. Examples of Measurement Results

As explained above, the 2-D line profiler allows simultaneous measurement of the time position of the photons reflected from the target points in 256 directions, in order to produce a line profile of the target under illumination with a spatial resolution of 256 pixels. The measurement results that follow demonstrate the performance of the 2-D line profiler under a variety of illumination conditions. The shown line profiles are recorded indoors and outdoors from interior and exterior concrete walls, i.e., from Lambertian-type targets (the reflection coefficient of a gray concrete wall is ~0.5).

#### 3.2.1. Indoor Measurements

**Figure 5:**
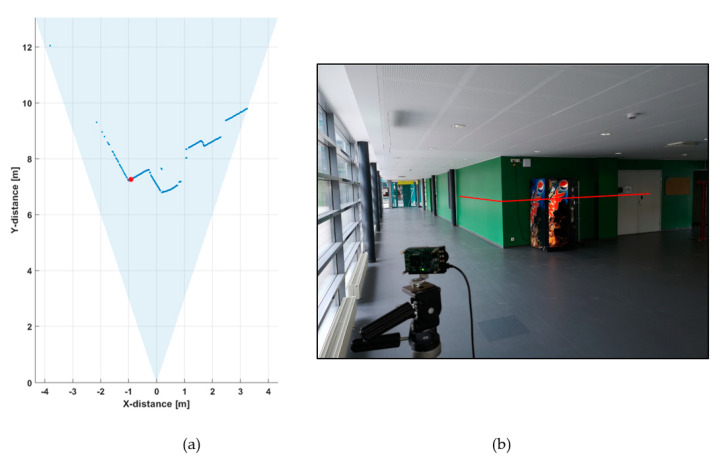
(**a**) 2-D line profile within the system field-of-view (FOV) and (**b**) photograph of the system FOV.

Figure 5a shows a line profile measured within a distance range of 1–12 m indoors at a low background illumination level of 200–1000 lux, depending on the position within the line profile. A photograph of the measurement scene is shown in Figure 5b.

**Figure 6:**
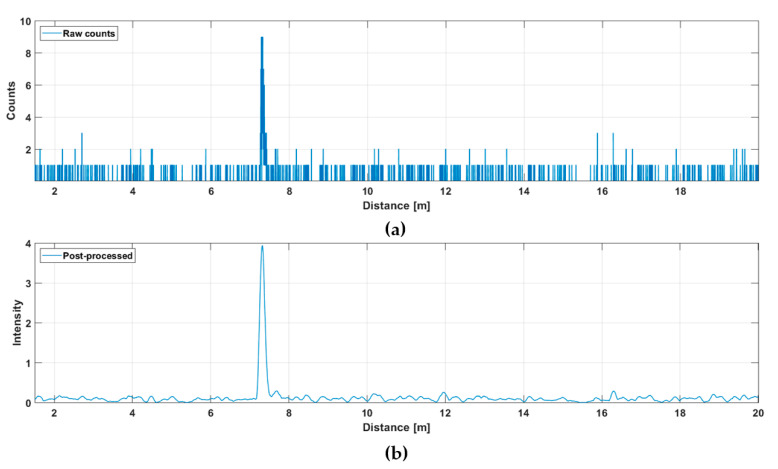
Hit distribution in one of the 256 SPAD channels: (**a**) raw counts and (**b**) filtered intensities.

This line profile was produced with 5000 laser shots, so that the corresponding line measurement rate at a laser pulsing rate of 130 kHz was 26 fr/s. The hit distribution for one of the 256 channels (marked with a red dot in Figure 5a) is shown in Figure 6, where the upper graph indicates the raw distribution of the recorded detections as a function of distance and the lower graph shows the corresponding distribution filtered with a triangular weighted running average filter with a FWHM width of 600 ps.

Before filtering, the raw detection histogram is also gain compensated. Since the SPADs are activated synchronously and only once per laser cycle, the histograms are distorted for high photon rates. For example, detections due to high level of background light are not uniformly distributed but follow an exponential distribution. The gain compensation aims to compensate for this distortion by noting, that detecting a photon at some time point after activating the SPAD is only possible if the SPAD has not already been triggered earlier. The probability, that the SPAD has been triggered before a certain time bin can be estimated by the sum of preceding histogram time bins. The gain compensated count for time bin i is then calculated by

(3) $$N_{i}^{\text{’}}=N_{i}\frac{N_{\mathrm{TOT}}}{N_{\mathrm{TOT}}-\sum_{j=1}^{i-1}N_{j}}$$

where $N_{i}^{\text{’}}$ is the gain compensated count, $N_{i}$ is the raw detection count and $N_{\mathrm{TOT}}$ is the total number of laser shots accumulated in the histogram, i.e., all time bin counts are divided by the estimated probability that the SPAD has not already been triggered earlier.

The distance result for the line profile image (as indicated in Figure 5a) is estimated by locating the histogram bin, which holds the highest count value in the post-processed histogram.

**Figure 7:**
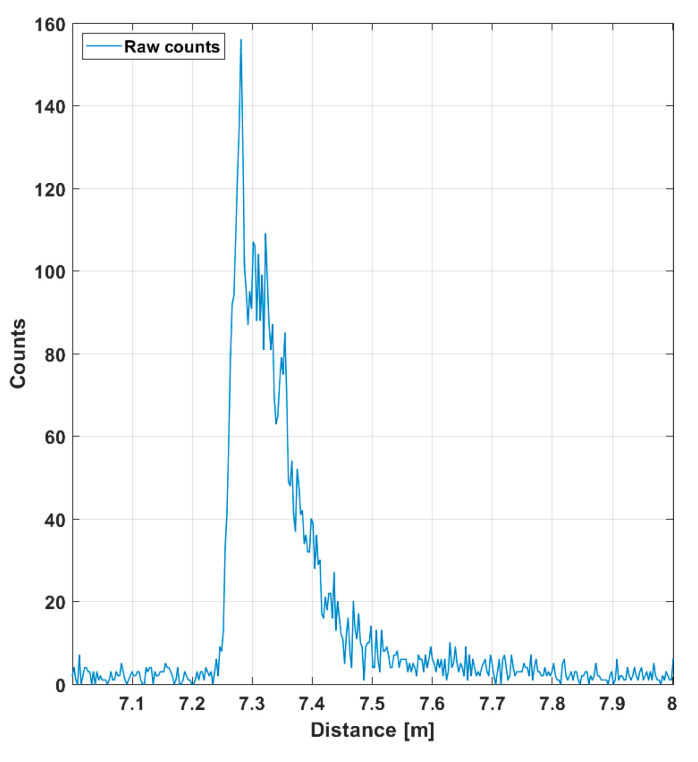
Echo intensity in one of the 256 SPAD channels (marked with a red spot in Figure 5a), while 130,000 laser shots (total duration 1 s) were used for the measurement. A total of 10 cm in distance corresponds to ~670 ps in time.

The SN_BG_R in the filtered histogram is quite high (~100), which shows up as good precision in the line profile results. The SN_BG_R is estimated here and in all subsequent similar plots as a ratio between the peak intensity, in this case ~4, and the sigma value of the variation in the number of background hits (variation in the line indicating the intensity of the background). The more detailed Figure 7 shows the shape of a detected pulse recorded with 130,000 laser shots (i.e., in 1 s), a shape that coincides quite well with that of the laser pulse, indicating that the jitter of the measurement system is in the sub-100 ps range. As can be seen, although the gain switching in the pulse is pronounced, it also has a relatively strong tail part, which is emphasized at a high driving current, especially with quantum well (QW) laser diodes working in the enhanced gain-switching regime [37]. The afterpulsing structure has practically no effect as such, but even shorter pulse (without reducing the energy) would be preferred. With bulk LDs, the pulse width (FWHM) can be decreased to ~100–150 ps depending on the laser cavity parameters.

#### 3.2.2. Outdoor Measurements in Moderate Background Illumination

**Figure 8:**
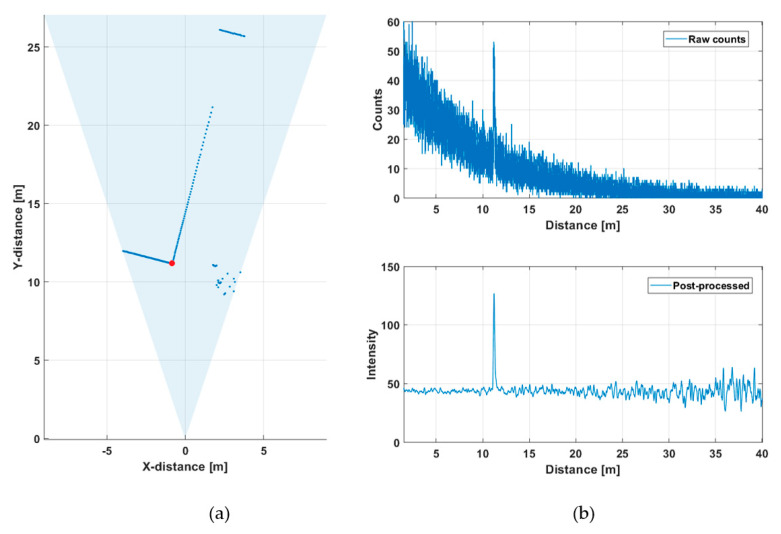
(**a**) Line profile of the scene presented in Figure 9a and (**b**) hit distribution in one of the 256 SPAD channels (marked with a red dot in Figure 8a); raw counts in the upper graph, post-processed intensities in the lower graph.

**Figure 9:**
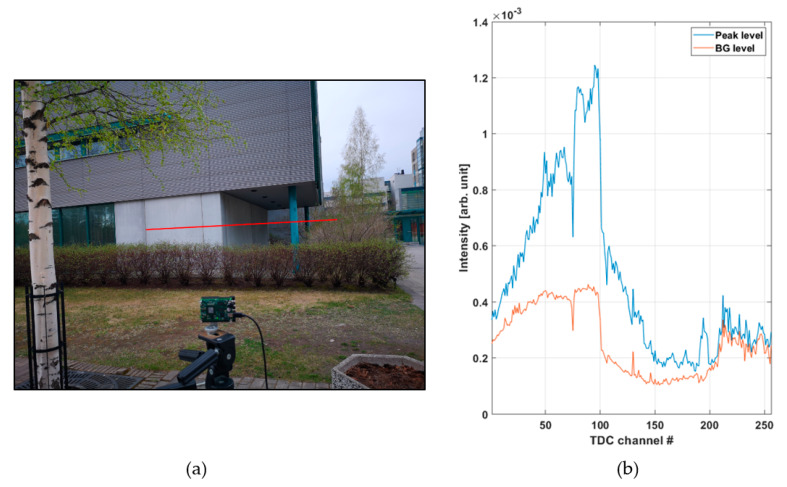
(**a**) Photograph of the outdoors measurement scene and (**b**) intensities of both the signal and background hits in 256 SPAD channels. The steep drop in measured intensity at around TDC channel ~100 corresponds to the corner of the building in (a).

Figure 8a shows a line profile measured within a distance range of 10–27 m outdoors at a background illumination level of ~15 klux (measured on the bright portion of the wall on a cloudy day, see Figure 9a) with a measurement time of 1 s (130,000 laser shots). Figure 8b presents recorded histograms of the raw hits and the post-processed (gain compensated and filtered) version which was used to calculate the results shown in the line profile. A photograph of the scene and the intensities of both the signal and background hits are shown in Figure 9a and Figure 9b, respectively.

Figure 8a shows the shape of the building quite clearly, and the supporting pole on the right side of the scene can be distinguished, as well as some echoes from the birch tree. A portion of a wall at a distance of ~27 m is also clearly recorded. The raw hit distribution in Figure 8b is not uniform since there was a considerable amount of background illumination during the measurement. The mean time interval between the background hits, determined from the time constant of the exponential distribution, is ~50 ns, which fits well with the measured background illumination and the system parameters (Equation (2)).

**Figure 10:**
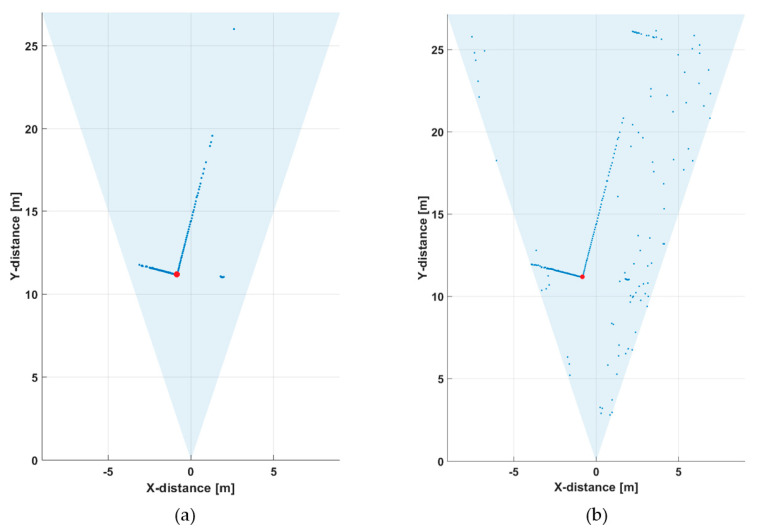
Line profiles measured at a line rate of 10 lines/s (13,000 laser shots) with two detection threshold settings: (**a**) ~2.5 × σ_BGnoise_ and (**b**) ~1.25 × σ_BGnoise_.

Figure 10a presents the same results when measured with a tenth of the number of laser pulses, i.e., at a line rate of 10 lines/s. As can be seen, some details (e.g., the echoes from the birch tree and the wall at ~22 m) are now missing from the profile, but the detection threshold was the same as in the previous measurement (the threshold level for a valid detection was ~2.5 × the sigma value of background intensity variation above the average value of the background, i.e., ~2.5 × σ_BGnoise_). Lowering of the threshold by a factor of two, in Figure 10b, meant that the details of the line profile could still be seen (as in Figure 8a), but at the cost of producing many random detections around the measurement scene.

**Figure 11:**
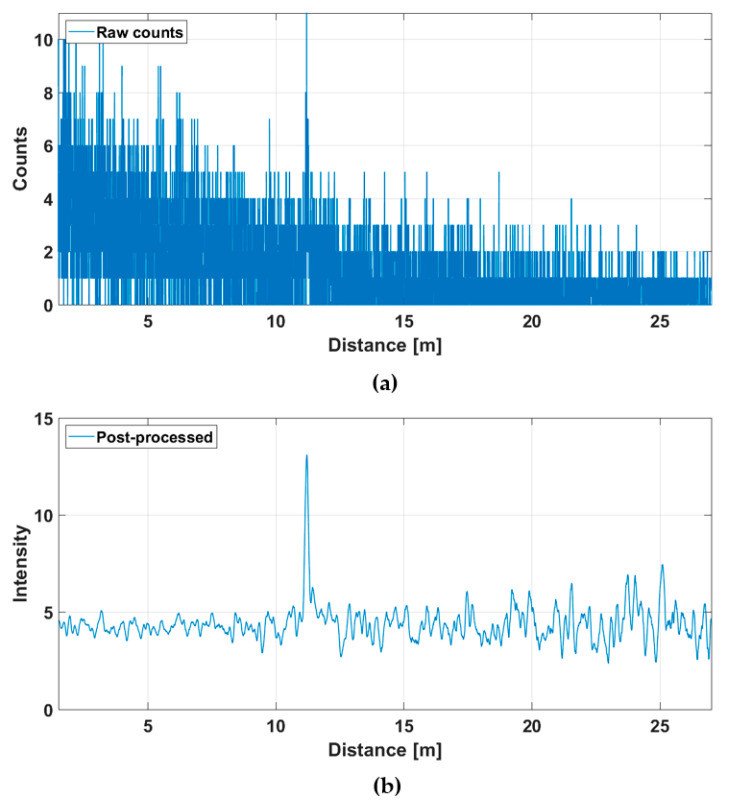
Hit distribution in one of the 256 SPAD channels in the line profile presented in Figure 10: (**a**) raw counts and (**b**) gain-compensated and filtered intensities.

Figure 11 presents the raw and post-processed (gain compensated and filtered) hit distributions for one of the SPAD/TDC channels (indicated with a red dot in the line profiles presented in Figure 10) when measured at a line rate of 10 lines/s. The hit distributions are noisier than those obtained with a measurement time of one second and the SN_BG_R (~30) at the target distance is approximately three times lower, as expected based on Equation (2).

#### 3.2.3. Outdoor Measurements in Strong Background Illumination with/without Gating

**Figure 12:**
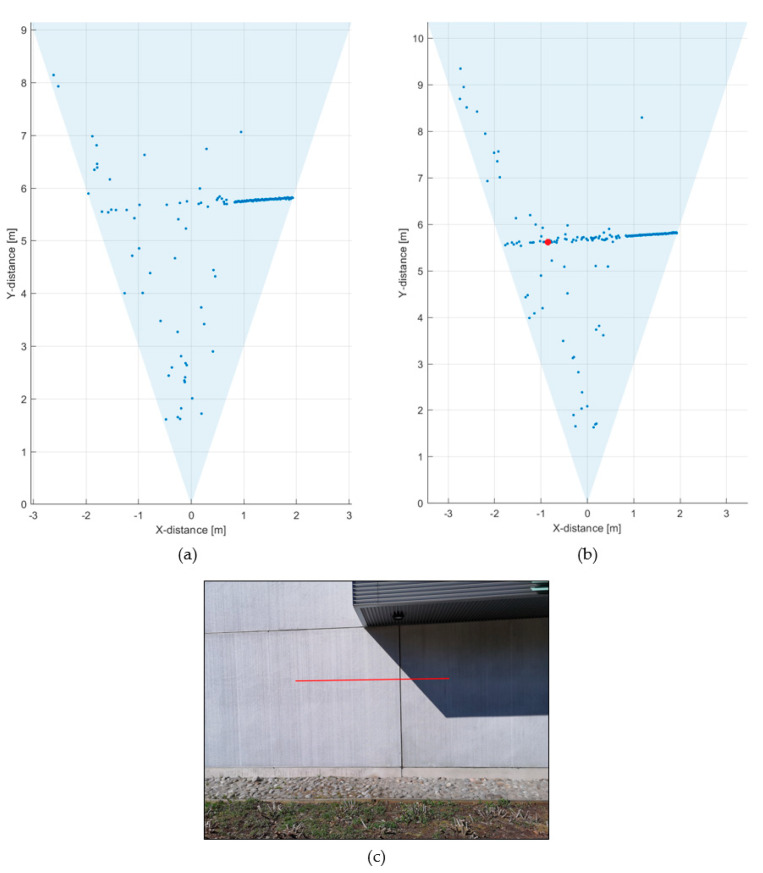
Line profiles of the scene measured with background illumination of 8k–70 klux: (**a**) at 5000 laser shots per pixel and (**b**) at 13,000 laser shots per pixel; and (**c**) photograph of the measured scene.

Another outdoor measurement was performed under sunny conditions, as shown in Figure 12, measuring two line profiles with 5000 (Figure 12a) and 13,000 (Figure 12b) laser shots, respectively. As can be seen, the measurements with 5000 laser shots (equivalent to 26 lines/s) were successful in the shaded area but only here and there at the given threshold level of 1.25 × σ_BGnoise_ in the area under direct sunlight (background illumination level ~70 klux). However, when the number of laser shots was increased to 13,000 per pixel (equivalent to 10 lines/s, Figure 12b), the whole line profile was already recognizable although there was still rather a lot of noise due to the low detection threshold. It was also found that, due to the lower SNR in the channel, the precision of the measurements was lower under the higher background illumination. The variation in the calculated distance results was ~5 cm, i.e., approximately 5 times greater than in the channels belonging to the shaded area (where the variation was <1 cm, corresponding to the roughness of the concrete wall).

**Figure 13:**
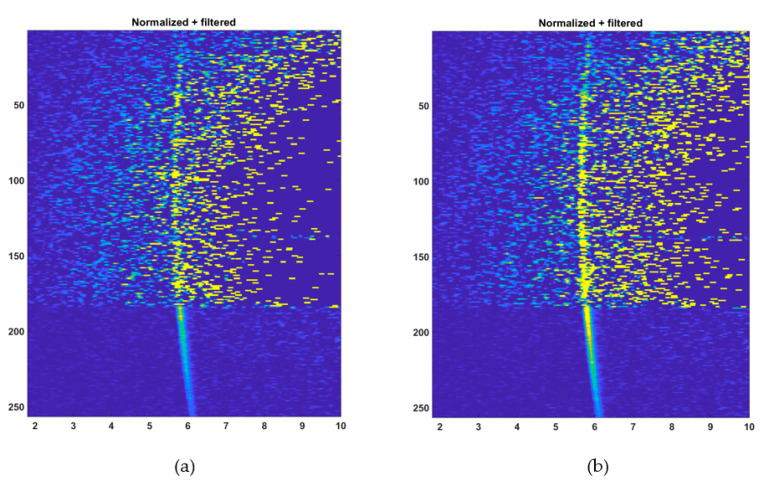
Normalized and filtered count intensity in 256 SPAD/TDC channels as a function of distance: (**a**) at 5000 laser shots per pixel and (**b**) at 13,000 laser shots per pixel. *y*-axis indicates the index of the SPAD/TDC-channel and *x*-axis the distance in meters. The number of detections is indicated by the brightness of the color.

It is perhaps also illustrative to look at the hit histograms in all the SPAD/TDC channels as a function of distance. These are shown in Figure 13. In this figure, the *y*-axis indicates the number of the TDC channel and *x*-axis the distribution of measured distance measurement results for the given number of laser shots. The brightness of the markings is proportional to the relative number of results corresponding to the location of the marking. The histograms concerned were produced from the raw counts after gain compensation and filtering, but before applying the coordinate transform. The wall is shown slightly curved, because *x*- and *y*-axis now represent the measured distance and the TDC channel index, rather than x-distance and y-distance coordinates. Both the histograms reveal the position of the wall quite clearly, even that with 5000 laser shots, although the SNR at the pixel obtained under high background illumination is quite low. These results suggest that spatial filtering (not applied here) could potentially be used to improve the image quality.

**Figure 14:**
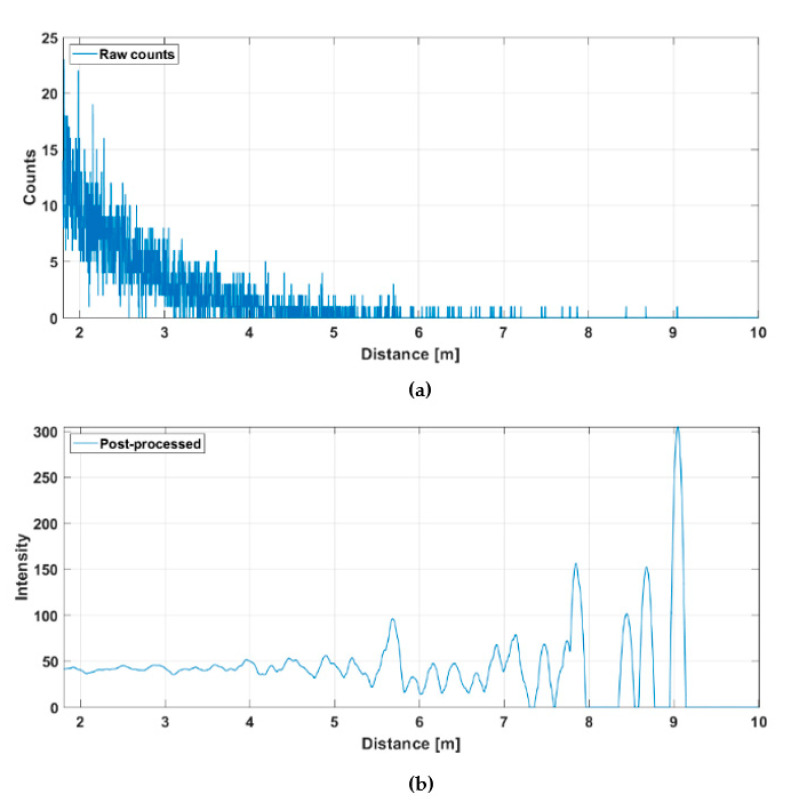
Hit distribution in one of the 256 SPAD/TDC channels with a measurement rate of 10 lines/s presented in Figure 12b: (**a**) raw counts and (**b**) gain compensated and filtered intensities.

Figure 14 shows the hit distribution of one of the 256 channels (marked with a red dot in Figure 12b) measured at a rate of 10 lines/s. The upper graph indicates the raw distribution of the recorded detections as a function of distance and the lower graph shows the distribution after gain compensation and application of a running averaging filter (FWHM = 600 ps). It is seen that while the raw hit distribution in Figure 14a barely allows the detection of any signal hits, these are easily recognizable in the post-processed intensity distribution. The SN_BG_R in the post-processed histogram of the SPAD/TDC channel shown in Figure 14b is ~7 at the target distance of ~5.5 m. Note also the clear deterioration in SN_BG_R as a function of distance. The time constant of the exponential hit distribution is ~7 ns, which corresponds to the measured background illumination level, the system parameters and the relations given in Equation (2).

The intensive background radiation in the measurements recorded in Figure 12 caused powerful blocking of the detected hits at the target distance (suppression ~0.005) and reduced the SN_BG_R by a factor of >10. This is a property of the SPAD/TDC detector configuration used here, which enables only one triggering of a photon per emitted laser pulse and thus imposes a serious limitation on the performance of the system. One way of alleviating this is to use electronic gating to open the SPAD detectors for the active detection mode only after a certain time delay with respect to the laser shot. The present system allows time gating with a resolution of 20 ns.

**Figure 15:**
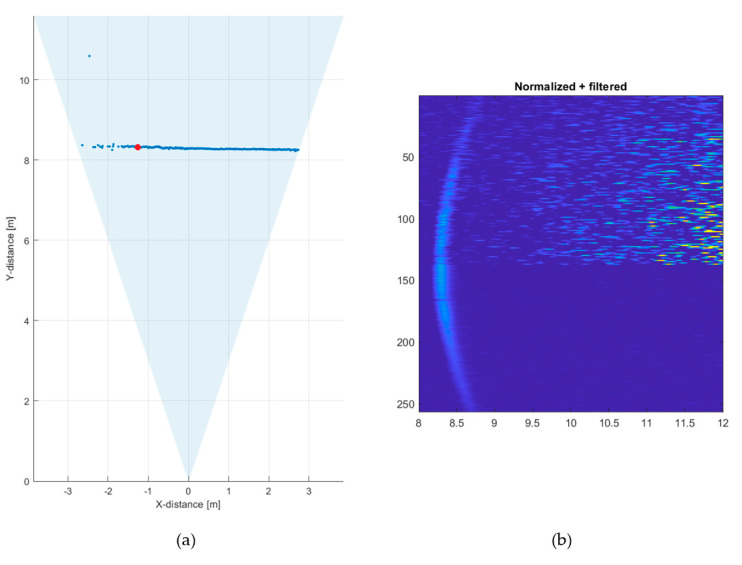
(**a**) Line profile measured at 70 klux/10 klux at a rate of 10 lines/s with opening of the SPADs for the detection mode ~40 ns after the laser shot and (**b**) corresponding intensity map for all 256 SPAD/TDC channels. 70 klux/10 klux corresponds here to the measured background illumination levels under the part of target that was in the direct sunlight and in the shadow, respectively, see Figure 12.

To demonstrate the effect of such gating, the gate was set to open ~40 ns after the laser shot. This allowed the maximum range to be increased to ~8.5 m under the above conditions. The corresponding measured line profile and the normalized and filtered count intensities in the 256 SPAD/TDC channels as a function of distance when measured at a line rate of 10 lines/s are shown in Figure 15. It is seen that the quality of the line profile is better than what was achieved without the gating at the shorter distance of ~5.5 m, although some results to the left of the line are still missing.

#### 3.2.4. Measurements in Strong Background Illumination with Varying Receiver Aperture Sizes

**Figure 16:**
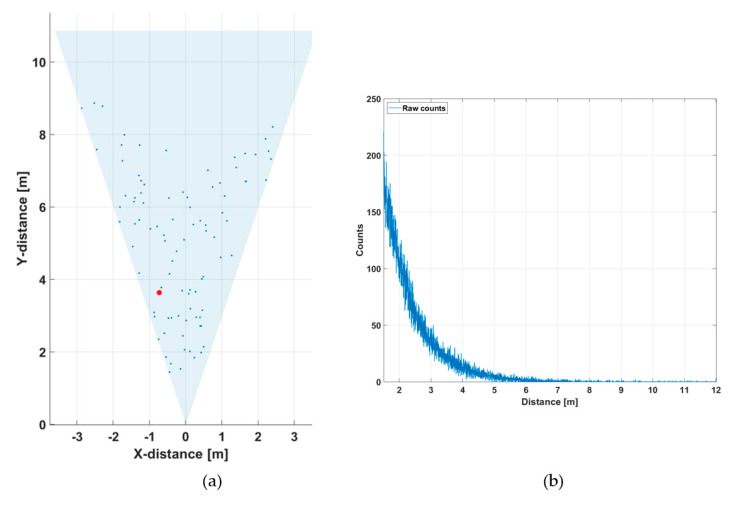
(**a**) Line profile of the scene presented in Figure 12, measured at 90 klux with an f/1.4 aperture, measurement rate 10 lines/s and (**b**) hit distribution in one of the SPAD/TDC channels, measurement time 1 s.

To demonstrate the effect of reducing the receiver aperture in improving the SN_BG_R and quality of the line profile measurement results, further measurements were carried out at the same outdoor location but on a different day and at a different time of day, so that the background illumination level was not exactly the same. During these measurements, the background illumination level was ~90 klux at the target (a concrete wall located in direct sunlight along the whole measurement line profile). In the first measurement, the whole receiver aperture of 11 mm (diameter) was used. As may be seen from the line profile result in Figure 16a, the target wall, which was now at a distance of ~10 m, was not recognized at all at the measurement speed of 10 lines/s due to the large number of random background photons. The hit distribution of one of the 256 SPAD/TDC channels is shown in Figure 16b. The measurement time for the distribution was 1 s in this particular measurement (Figure 16b), so that the time dependence of the distribution could be better resolved. A pronounced attenuation with a time constant of 5–6 ns is seen in the number of recorded counts, which corresponds well with the measured illumination level and system parameters.

**Figure 17:**
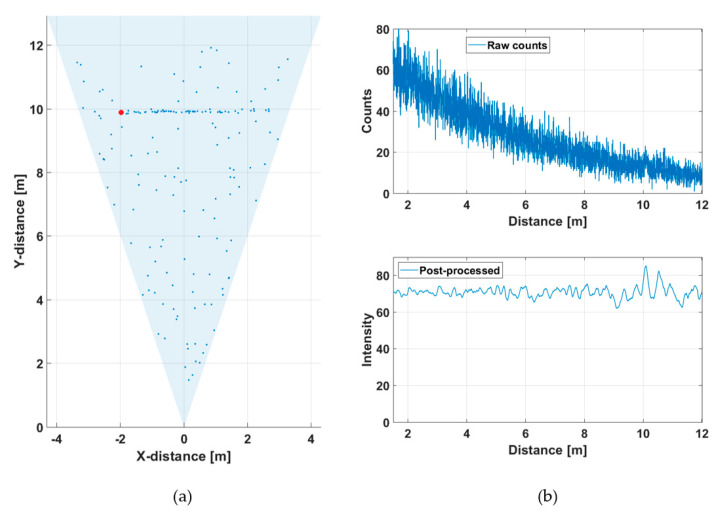
(**a**) Line profile of the scene presented in Figure 12, measured at 90 klux with an f/4 aperture, measurement rate 10 lines/s and (**b**) hit distribution in one of the SPAD/TDC channels, measurement time 1 s.

If the aperture is reduced to ~4 mm, however, the SN_BG_R is improved and the target is now recognizable, especially in the center portion of the system FOV, see Figure 17a. This results from the fact that, although the SN_BG_R is in principle lower due to the smaller aperture, the blocking effect is reduced even more, and thus the overall SN_BG_R improves, as suggested by Equation (2). The hit distribution shows a markedly longer time constant of 40–50 ns (and thus lower blocking), as could be expected on account of the reduced aperture.

**Figure 18:**
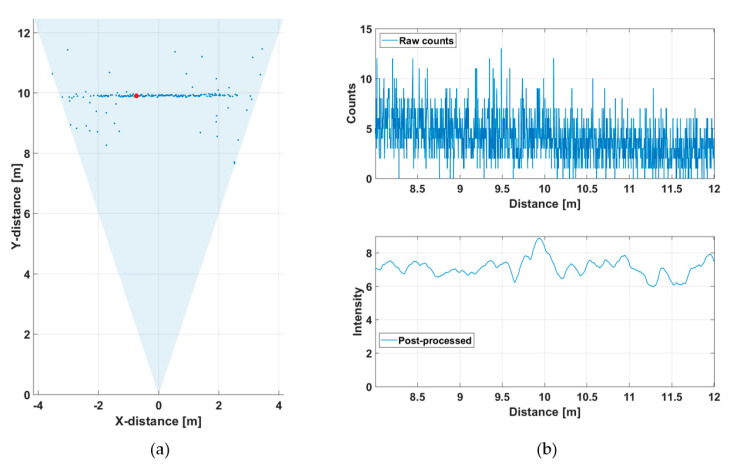
(**a**) Line profile measured at 90 klux with an f/4 aperture and time gate delay of 40 ns, measurement rate 10 lines/s and (**b**) hit distribution in one of the SPAD/TDC channels, measurement time 10 lines/s (0.1 s).

A further improvement in the line profile recording was achieved by applying time gating to the SPADs, so that they were activated ~40 ns after the laser shot transmission. The line profile measured at a line rate of 10 line/s is shown in Figure 18a. The target is now quite well resolved, although there are also random hits at the edges of the FOV due to the lower signal intensity in that area.

The hit distribution of one of the 256 SPAD/TDC channels (marked with a red dot in Figure 18a) is shown in Figure 18b as measured with a line rate of 10 lines/s (i.e., 0.1 s). It is seen that the simple filtering and detection algorithm used enables one to find the correct distance result from relatively noisy raw data.

## 4. Discussion and Summary

An analysis is presented here of the relation between signal and noise strengths in SPAD-based pulsed time-of-flight 3D range imaging, especially with respect to measurement conditions limited by background noise and on the assumption that the probability of a single emitted laser pulse being detected in any given SPAD detector is <1. In addition, several practical examples of measurements using a solid-state pulsed TOF line profiler were presented and analyzed in the light of the existing theory.

In particular, the importance of the mean time interval between the random detections induced by the background illumination (τ_BG_) for the performance of the system was demonstrated by means of practical measurements performed under different illumination conditions. To optimize the system performance, it is important to make this time interval as long as possible, as this will decrease the noise induced by the background illumination, which is the dominant source of noise in many practical instances. In addition, this will minimize the blocking effect, which is otherwise hard to regulate without markedly increasing the complexity of the system, i.e., by using re-triggerable, actively quenched SPADs and multichannel TDCs per pixel. In practice, the blocking effect shows up seriously when the time interval between the random detections induced by the background illumination (τ_BG_) is comparable to the flight time of the photons scattered from the target (2 R/c), as indicated by the exponential term in Equation (2). The effective means of increasing τ_BG_ involves reducing the optical bandwidth of the illumination, and especially the field-of-view of the SPAD element. These are not “free parameters”, however, since their selection will affect the other system parameters as well. Reduction of the optical bandwidth by using DBR laser diodes, for example, would lead to a decrease in laser pulse power, and the FOV of a SPAD element affects the FOVs of the whole system. Thus, careful optimization according to the needs of the specific application is needed at the system level.

Coincidence detection has also been suggested as an approach to decrease of the effect of the background illumination, see e.g., [41,42,43] and references therein. A recently published SPAD-based line profiler with 2 × 192 pixels utilizing adaptive photon coincidence detection demonstrates distance measurement results in bright light at a distance of ~7 m but at the cost of markedly higher pulse and average powers than what were used in this work [43]. In principle, coincidence detection would reduce the probability of noise triggering and thus the exponential blocking, when SPAD is operated synchronously with the laser and only one detection is allowed per pulse. However, in practice, the coincidence detection is challenging due to lack of simultaneous photons (within one pulse envelope), especially at the limit of sensitivity, i.e., near the maximum range.

The main conclusion of the work is that in general it is advantageous to concentrate the available (or permissible) average optical power in short, intensive pulses and to focus the optical energy in spatial terms. The focusing of the optical energy could be achieved in 3-D range imaging by replacing the regularly used flood illumination with block-based illumination [28]. Block-based illumination can be realized by means of a laser diode bar (e.g., consisting of 16 element laser diode bar) and a cylinder lens system or a holographic diffuser. Only one of the laser diodes (or a nano-stacked LD) within the LD bar would then be driven per single emitted pulse with all available/allowed drive energy. In another realization option, the FOV of the system is illuminated with a vertical cavity surface emitting laser (VCSEL) laser diode that would include an array of individually addressable sub-elements (which are 2D arrays themselves, homogenized e.g., with a diffuser). Now, only one of these separate VCSELs (in the 2D VCSEL array) is driven per an emitted laser pulse. Thus, the maximum drive energy (pulse current) can be used to drive the specific VCSEL sub-element. The advantage of the addressable VCSEL array techniques is the simple optical realization since the dimensions of the VCSEL array and its elements can be scaled to correspond to the desired illumination patterns and thus simple optics can be utilized in the transmitter. On the other hand, with VCSELs it is hard to go to sub-ns pulse regime, which then trades off with the precision. Another VCSEL transmitter approach would be to use the array properties of the VCSEL array to directly define the spatial resolution of the measurement (rather than the SPAD receiver) as in [44].

**Figure 19:**
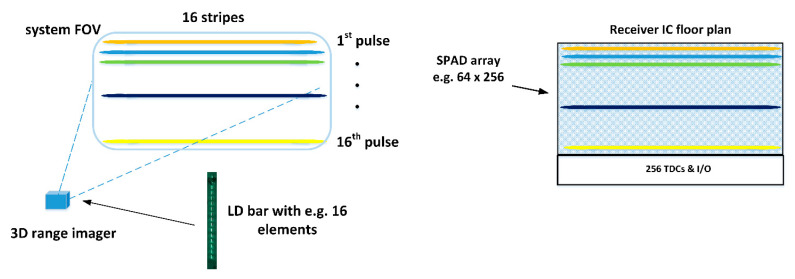
The block-based illumination concept. The transmitter uses illumination blocks or stripes (as shown here) and the receiver consists of a uniform SPAD array and a separate TDC bank, which can be connected to any of the illuminated SPAD rows/blocks.

One modification of the LD-bar approach could be an illuminator that would illuminate the system FOV with narrow laser stripes. In this case, the vertical and horizontal resolutions would be defined by the number of stripes and the number of SPAD elements in a row, respectively, as depicted in Figure 19, i.e., the proposed system would be a kind of generalization of the line profiler presented in this paper. The illuminator could be based on a sequentially driven laser diode bar, for example, i.e., in fully solid-state realization [28]. In this case, it would be appealing because of the easier opto-mechanical adjustments involved to realize the SPAD receiver with a dense 2D array and then select electrically only those rows for operation that actually receive laser energy. The use of narrow stripes in the illumination would increase the irradiance on the target, and thus, as indicated by Equation (2), also the SN_BG_R, albeit at the cost of reduced vertical resolution. This type of transmitter approach seems to be suggested recently also in [45].

The line profiler used here functioned by means of a custom-designed laser diode working in the “enhanced gain switching” mode, producing sub-ns laser pulses with an energy of ~2 nJ. Thus, the average optical power of the illuminator is ~260 µW at the pulsing rate of 130 kHz. It should be noted, however, that an increase in the measurement range to some tens of meters in bright sunlight would require considerably more laser energy. A laser pulse width of ~1 ns and energy of some 20–40 nJ would seem to be feasible with current laser driver techniques, and this would markedly improve the system performance, especially if the blocking effect could be diminished by proper system parameter selections and by time gating, for example.

## Figures and Tables

**Table 1:** Key parameters of the developed 2D line profiler.

Transmitter			SPAD/TDC Receiver				
Mean Ill. Power	Wavelength	FOV	SPAD Array	SPAD Pitch/FF	TDC Array	TDC Depth/reso.	Technology
0.26 mW@130 kHz	810 nm	37° × 0.3°	256 × 1(8)	41.6 µm/35%	256 × 1	15 bits/19.5 ps	0.35 µm CMOS
